# The U-Shaped Curve of Health Inequalities Over the 20th and 21st Centuries

**DOI:** 10.1177/27551938241244695

**Published:** 2024-04-01

**Authors:** Clare Bambra

**Affiliations:** Population Health Sciences Institute, 12186 Faculty of Medical Sciences, Newcastle University, Newcastle upon Tyne, UK

**Keywords:** social inequality, history, public health policy, income inequality, social determinants of health

## Abstract

This article examines historical trends in health inequalities over the 20th and 21st centuries. Drawing on studies from the United States, United Kingdom, Sweden, and Western Europe, it concludes that there is evidence of a u-shaped curve in (relative) health inequalities. These trends in health inequalities broadly parallel those identified by economists with regards to the u-shaped curve of income and wealth inequalities across the 20th and 21st centuries. The article argues that—as with income inequalities—health inequalities generally decreased across the twentieth century through to the early 1980s. They then started to increase and accelerated further from 2010, particularly in the United Kingdom and the United States. The article sets out four distinct policy periods that shaped the evolution of trends in health inequalities: the Interbellum Era, 1920–1950; the Trente Glorieuse, 1950–1980; Neoliberalism, 1980–2010; and the Crisis Age, 2010–present. The u-shaped curve of health inequalities over this period suggests that social policies, health care access, and political incorporation have driven changes over time. Taking this long view of changes in health inequalities emphasizes the importance of politics and policy for future health improvement.

In his seminal work, *Capital in the Twenty-First Century*, the French economist Thomas Piketty identified a u-shaped curve in wealth and income inequality over the 20th and 21st centuries.^1^ Given the high profile, parallel discussion about the relationship between income inequality and health,^2^ this article draws on historical epidemiological studies to argue that there is a similar u-shaped curve in health inequalities. Whilst evidence from the early part of the twentieth century is sparse, the historical data available from the United States, Sweden and the United Kingdom suggests declines in health inequalities from the 1920s to the 1950s.^3–6^ A larger body of work from the United States, the United Kingdom, and Western Europe examines trends from the 1960s to the early twenty-first century.^7–18^ Overall, these studies find that health inequalities further reduced from the mid-1960s until the early 1980s when they increased again—with an acceleration in the pace of increase in the early part of the twenty-first century. This article documents this u-shaped curve of health inequalities over the 20th and 21st centuries, examines potential explanations of the policies and politics that lie behind the curve, and uses these to identify how we can break the curve in the future.

## Wealth and Income Inequality in the 20th and 21st Centuries

Using novel data sources, Piketty calculated the share of total national income and wealth held by the top decile of the income distribution (the top 10%) and the top percentile (the top 1%) across high-income countries.^1^ This built on earlier work by Piketty and Saez that had examined similar trends in the United States^19^ where they identified a u-shaped curve of wealth and income inequality over the 20th and 21st centuries. Between 1920 and 1929 in the United States, the share of national income taken by the top 10 percent rose from around 40 percent in 1920 to 50 percent in 1929. It then fell sharply following the Wall Street Crash of 1929 and then again during World War II, stabilizing at around 35 percent from the mid-1940s to the mid-1970s. It then rose rapidly through the 1980s, peaking again at 50 percent in 2007. A similar pattern is evident for the share of national income held by the top 1 percent in the United States (peaking at around 24% in both 1929 and 2007).^1,19^

These temporal changes in the proportion of income and wealth held by the top groups are also evident for the other “Anglo-Saxon” countries with 2007 peaks of a 10 percent share of income for the top 1 percent in Australia, 14 percent in Canada, and 15 percent in the United Kingdom.^1^ The national share of income held by the highest groups also increased from the 1980s in the other wealthy countries of Japan and Western Europe (e.g., Denmark, France, Germany, Italy, Spain, and Sweden) although from a lower base and with a smaller peak. For example, in France, Japan, Italy, and Spain, the share of the top 1 percent increased from approximately 7 percent in 1980 to 9 percent in the 2010s; in Sweden, from 4 percent to 7 percent; Denmark, from 5 percent to 7 percent; and Germany, from 9 percent to 11 percent. As Piketty notes, “their trajectory resembles that of the United States in some respects, with a delay of one or two decades”.^1^, p. 321. Subsequent analysis has found that the higher shares of national income held by the top 10 percent and top 1 percent have been maintained since 2010.^20^

## The U-Shaped Curve of Health Inequalities

This section examines what is known from historical epidemiological studies about trends in health inequalities over the 20th and 21st centuries. Data on early twentieth-century trends in health inequalities is available in studies of infant mortality rates (IMRs) in the United States and Sweden^3,4,5,6^. Overall, IMRs fell dramatically over the twentieth century for all social groups. For example, in the United States, IMRs fell from an average of 61 per 1,000 live births in 1935 to six per 1,000 live births in 2020, and in Sweden from 50 to two.^21,22^ However, studies have noted a “fall and rise” of inequalities in IMRs in the United States over the twentieth century.^7^ For example, Rodriguez and colleagues, in a study of state-level trends in IMRs from 1925 to 2017, found that inequalities by race/ethnicity in the United States declined between 1925 and 1945, then increased slightly until the mid-1960s, then declined again until around 1980 when they increased until 2017 (with a period of slight decrease from around 2000 to 2010)^3^. A similar pattern has been found in analysis of historical trends in inequalities in IMRs for Sweden. Socio-economic inequalities in IMRs in Sweden first appeared in the late 1890s^23^ and then decreased steadily over most of the twentieth century through to 1980 when they were at their lowest.^4^ In the early- to mid-1980s, socioeconomic inequalities in IMRs in Sweden then increased between the most and least privileged groups.^9^

Research examining more recent historical trends in inequalities in IMRs is available for the United States, Sweden, Norway, Denmark and England. Analysis by Krieger and colleagues^7^ of county-level trends in IMRs in the United States between 1960 and 2002 found that absolute and relative racial/ethnic inequalities in IMRs fell between 1966 and 1980 (and particularly between 1965 and 1971)^8^ but that relative inequalities rose again between 1980 and 2002 while absolute inequalities stagnated.^7^ They also found similar trends for income inequalities in IMRs in the United States—shrinking relative and absolute inequalities between the top and bottom quintiles of income before 1980, followed by their widening or stagnating thereafter.^7^ Relative inequalities in IMRs also increased in Norway and Sweden between 1980 and 2001, and in Denmark absolute inequalities increased in IMRs during this period.^10^ Analysis of socio-economic trends in IMRs in England from 1983 to 2017 also found that from the early 1980s to the late 1990s, absolute and relative inequalities in IMRs increased; there was then a slight decline in inequalities in the early part of the twenty-first century (1999–2010) before inequalities increased again from 2011 to 2017.^11^

This u-shaped curve is also evident in terms of trends in inequalities in premature and all-cause mortality in England and Wales, the United States, France and other European countries. Analysis by Thomas and colleagues (2010) of trends in socio-economic area-level deprivation in under-65 mortality in England from 1921 to 2007 found that the relative index of inequality in standardized mortality ratios (SMR) declined from a ratio of 2.50 in 1921–1930, through to a low of 1.92 in the early 1970s.^6^ From the 1980s onwards, it increased consistently rising to 2.79 in the mid-2000s. They found the same pattern for the SMR of best to worst of the most deprived 10 percent to the least deprived 10 percent.^6^ Likewise, work by Wilkinson also noted a u-shaped curve with a decrease in the slope index of relative occupational class inequalities in working-age SMRs among men in England and Wales between 1921 and 1951, albeit with slight increases from 1961 to 1971.^5^ Trends for women paralleled those for men from 1931, but with a less steep gradient.^5^

A similar trend was identified by Krieger and colleagues’ research in the United States.^7^ They found that inequalities by income and race/ethnicity in premature mortality rates (under 65 years of age) also present as a u-shaped curve with absolute and relative inequalities falling between 1966 and 1980, and then relative inequalities rising—and absolute inequalities stagnating—from 1980 onwards.^7^

Analysis of trends in educational inequalities in France in premature all-cause mortality rates (for men and women aged 30–64) from 1968 to 1996 also noted increases from the 1980s; the relative index of inequality increased from 1.96 (men) and 1.87 (women) in 1968–1974 to 2.77 (men) and 2.53 (women) in 1990–1996.^12^ The rise in health inequalities since the 1980s is also evident in other European countries. For example, Mackenbach and colleagues’ analysis of trends in relative educational inequalities in all-cause mortality for seventeen European countries from 1980 to 2014 found that while mortality rates declined steadily amongst all educational groups over this period, relative inequalities increased considerably.^13^

Looking into the early twenty-first century, evidence from the United States and the United Kingdom shows a further increase in health inequalities—to such an extent that they may be leading to a stall in overall health improvement.^14–18^ Prominent research in mortality trends between 1999 and 2013 in the United States by Case and Deaton found an increase in all-cause mortality among middle-aged white non-Hispanic people.^16^ These increases were concentrated among those without a 4-year college or bachelor's degree.^17^ Since 2010, death rates have also risen among Black non-Hispanic people and Hispanics without a degree.^17^ Overall, adult life expectancy in the United States over the last decade has risen for the college educated and fallen for the rest.^18^ Similarly, analysis by Marmot and colleagues^14,15^ of trends in inequalities in life expectancy in the United Kingdom has found that “over the decade since 2010, the social gradient in life expectancy has become steeper and the inequalities by area-level deprivation greater”.^15^ So, this recent increase in health inequalities in the United States and the United Kingdom is due to stagnation or declines in lower socioeconomic groups alongside continued improvement in higher groups.^15^

In summary, taken together, the historical studies reviewed here suggest that there is a u-shaped curve of health inequalities over the 20th and 21st centuries. For IMR, there is a u-shaped curve in relative inequalities—particularly evident from the 1960s onwards—in Sweden, the United States, and the United Kingdom. This curve is also evident—albeit to a lesser extent—in studies of relative inequalities in premature mortality in the United States, England, France and for all-cause mortality in England and Wales and continental Europe. There is also more recent evidence from the United States and the United Kingdom of an acceleration in the increase in health inequalities in the early part of the twenty-first century.

## Behind the Curve

**Figure 1.**
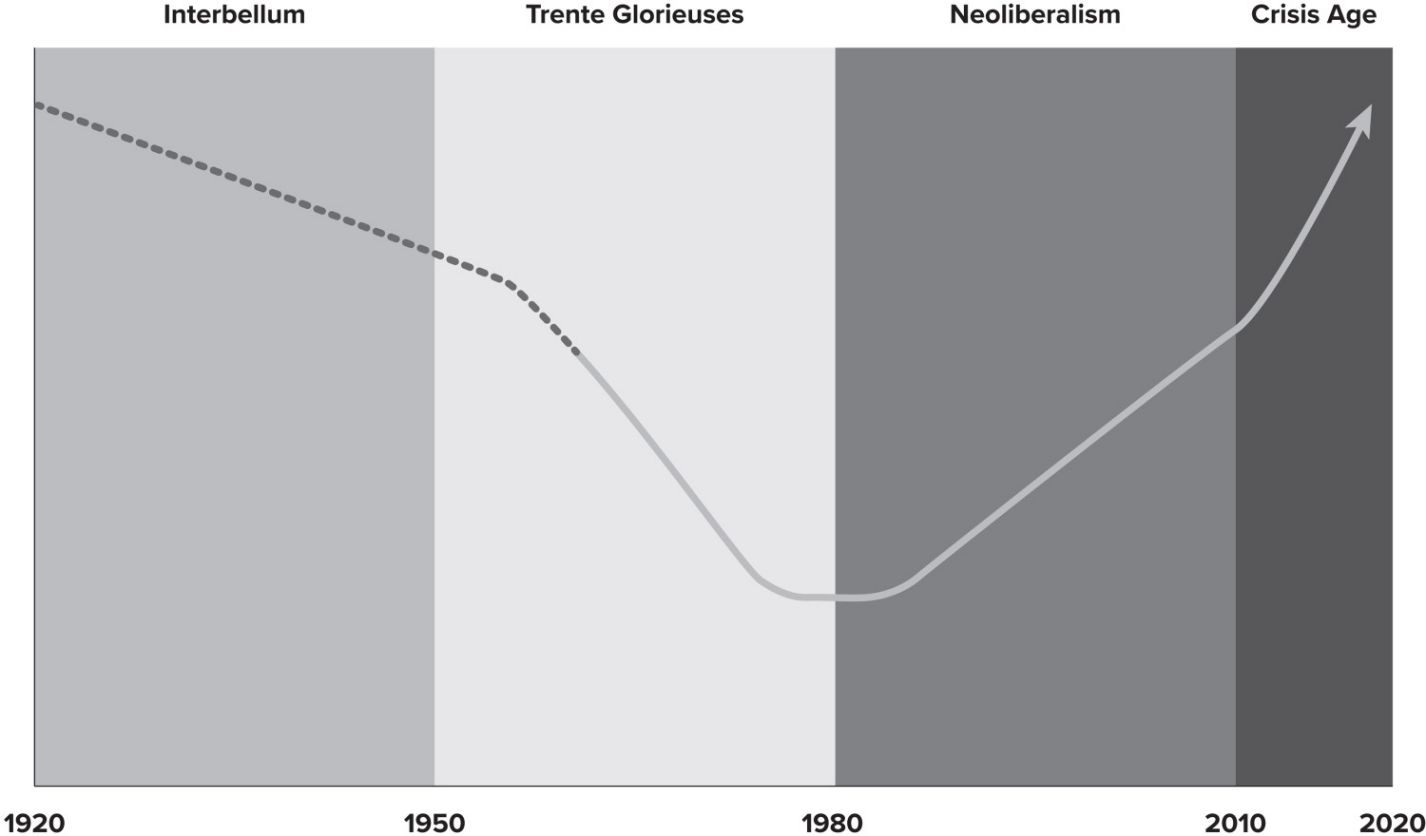
The U shaped curve of health inequalities over the 20th and 21st centuries.

These trends in health inequalities echo what Piketty^1^ and other economists, including Goldin and Margo^23^ and Krugman,^24^ have found regarding inequalities in wealth and income: decreases in inequality in the mid-twentieth century (what Goldin and Margo term the “Great Compression” in relation to income and wealth in the United States) then widening again, from the 1980s, to levels higher than those in the 1920s. This parallel between long-term trends in wealth and income inequality and trends in health inequalities are suggestive of an association between the two.^5^ The role of income inequality and *between* international inequalities in health and wellbeing between countries has been extensively set out by Pickett and Wilkinson (e.g., that more equal countries have better overall life expectancy)^2^. However, there has been less attention paid to the role that income inequality might have for health inequalities within countries (a notable exception being Wilkinson)^5^ and the limited analysis that has been undertaken to date has been somewhat inconclusive.^25^ The overview of historical trends in both income and health inequalities presented here suggests that there could be an association over the longer term. This would need to be explored more systematically but income inequality might drive health inequality through various mechanisms including lower wages, higher rates of unemployment, lower taxation rates on capital (therefore less expenditure on public services and welfare support systems), and higher poverty rates.^5,26^ Indeed, the economist Paul Krugman notes that the expansion and contraction of the welfare state was the main driver of income inequalities over the twentieth century.^24^ This section examines chronologically how changing policy regimes might explain the u-shaped curves of income and health inequalities across the 20th and 21st centuries, identifying four key periods (see Figure 1): the Interbellum Era (1920–1950), the Trente Glorieuse (1950–1980), Neoliberalism (1980-2010), and the Crisis Age (2010–present).

The Interbellum Era—or between the wars—was a period of social reform with the beginnings of welfare state systems in many countries. In Sweden, for example, Burstrom describes how improvements in water, sanitation, hygiene, social housing, nutrition, family policies, and health care over the early part of the twentieth century in Sweden particularly benefitted children in the poorest families.^4,27^ Average incomes also increased in this period in many countries and poverty rates and income inequalities fell.^1,4,5^ Likewise in the United Kingdom, the 1920s and 1930s saw the introduction of pensions, slum clearances and the start of social housing as well as an increase in the coverage of community-based health insurance. Poverty rates also decreased substantially in the United Kingdom in this period because of improved social security benefits.^5^ Technical advances in medicine were also beneficial, including better understanding of infectious diseases and more vaccination programs.^4^ In the United States, income inequality also fell dramatically because of progressive taxation, stronger unions, strong economic growth, and wage regulation under Roosevelt's New Deal.^28^ The New Deal also entailed public works projects, protection for labor organizing, established minimum wages, funded a house-building program, and it introduced pensions, unemployment insurance, and the welfare program Aid to Dependent Children.^29^

The Trente Gloriuses refers to the sustained period of economic growth experienced in Europe (and other high-income countries) from the 1950s to the late 1970s. This was the “golden age” of welfare state capitalism, characterized by centralism, universalism, and active macroeconomic management by the state (Keynesian economics) with an interventionist fiscal policy, a large public sector, and a mixed economy, full (male) employment and high public expenditure, and the promotion of mass consumption via a redistributive tax and welfare system.^30^ There was also a mainstream political consensus in favor of the welfare state and the redistribution it effected.^26^ Western European countries experienced significant improvements to public housing, health care, and the other main social determinants of health.^30^ This meant that in the 1950s and 1960s, income and wealth inequalities were historically at their smallest, and poverty rates the lowest.^1,5,23,24^ Similarly, the United States in the 1960s saw the Great Society social reform program which enhanced public health care coverage, the civil rights acts—which outlawed racial discrimination (Jim Crow laws) and segregation in public services—and poverty was reduced through increasing the value of the state pension, higher wages, and expanding the scope of Aid for Dependent Children.^31,32^

This golden age of the welfare state effectively ended with the economic crisis of the 1970s and the rise of neoliberalism or market fundamentalism.^26^ The fundamental presuppositions of neoliberalism are that markets are the normal, natural, and preferable way of organizing the economy and society and that the primary function of state institutions and policies is to ensure the efficient functioning of markets and market outcomes.^26^ Initially in the Anglo-American countries (under Reagan and Thatcher administrations) but then more broadly (e.g., Kohl in West Germany), the political consensus of the post-war settlement between labor and capital broke down as governments started to follow monetarist theories and dismantle and restructure the interventionist Keynesian welfare state.^33^ The “reforms” were characterized by the privatization and marketization of state services and industries; the retrenchment of social security benefits and social housing; modified taxation arrangements (with a shift away from business taxation); restrictions on labor organizing; and the abandonment of the state's role in promoting full employment.^26^ Wages fell and income inequality, poverty rates and unemployment all increased.^26^

The Crisis Age started with the Global Financial Crisis (GFC) of 2007–2008, which resulted from a downturn in the United States housing market. The GFC led to a massive collapse in global financial markets, a huge rise in government debts, and increases in unemployment and poverty.^34^ The GFC was accompanied in many European countries (including the United Kingdom, Italy, Greece, Portugal, and Spain) by escalating public expenditure cuts.^35^ These austerity measures entailed large scale cuts to public services, including health care, and steep reductions in welfare benefits for the poorest groups.^35^ Income and wealth inequality increased because of stagnating wages and higher poverty rates and tax cuts for the wealthy and corporations.^1^ The period since 2007–2008 has been a time of permanent crisis for high-income countries with simultaneous instability across political, economic, environmental, and global health systems. The crises include threats to democracy (with the rise of the populist and far right) and rolling wars in Europe and the Middle East; the return of protectionism (and a seemingly new “cold war” with China); environmental disasters and the impacts of climate change more commonplace and widespread (with, for example, the highest ever recorded temperatures in Europe and China in summer 2022); and the COVID-19 pandemic resulting in over seven million recorded deaths, and leading to unprecedented social and economic upheaval across the world.^15^ The latter has since turned into a post-pandemic cost-of-living crisis with rising inflation, interest rates, and unemployment. Poverty rates and income and wealth inequalities have increased in many countries in this period.^20^

## Breaking the Curve

Looking back over the 20th and 21st centuries and these four distinct policy periods, the Trente Gloriuese appears as an historic exception when income, wealth, and health inequalities were all at their lowest.^1,5,19^ This era teaches us that to break the u-shaped curve and reduce health inequalities again, large-scale policy action across all aspects of society is required. Previous research has identified three main mechanisms for levelling health inequalities in this period: poverty reduction through a redistributive welfare system, improved healthcare access, and enhanced democracy through the political incorporation of the working classes and marginalized groups.^36^ These levelling mechanisms work together to improve the health situation of the poorest in society—historically, democratization has tended to result in increases in welfare state and health care provision.^37^ This is particularly evident in the United States in the 1960s, for example, when the expansion of social security safety nets (and the reduction of poverty) was accompanied by increased health care access, especially for Black Americans as enabled by the Civil Rights Act of 1965.^8^

This analysis of the driving mechanisms behind the post-war reductions in health inequalities, is further reinforced from what can be seen as “blips in the curve” —reductions in health inequalities in Germany in the 1990s and England in the 2000s despite wider contextual trends of increasing income and wealth inequalities.^35,36^ The fall of Communism and the reunification of Germany in the 1990s provides an example of how to reduce health inequalities—significantly, at scale, and in a fairly short time frame. In 1990, the life expectancy gap between the former East and the former West of Germany was almost three years for women and three and a half years for men. This gap rapidly narrowed in the following decades so that by 2010 it had dwindled to just a few months for women and just over one year for men.^35,36,38^ This was achieved through similar mechanisms as the Trente Gloriuese: political incorporation through democratization in the East, improvements in the incomes of the poorest as well as better quality health care provision—all made possible by redistributive taxation.^35,38–41^

The English Health Inequalities Strategy in the 2000s is another example. This was a wide-ranging and multi-faceted health inequalities reduction strategy in which policymakers systematically and explicitly attempted to reduce inequalities in health.^42^ The cross-government strategy focused specifically on supporting families, engaging communities in tackling deprivation, improving prevention, increasing access to health care, reducing child and pensioner poverty rates, and tackling the underlying social determinants of health.^42^ These policies led to reductions in social inequalities in the key social determinants of health, including unemployment, child poverty, housing quality, access to health care, and educational attainment.^35^ These were accompanied by reductions in health inequalities between the most deprived areas in England and the rest of the country: inequalities in life expectancy decreased by just over a year for men and around six months for women;^43^ the gap in IMRs narrowed by 12 deaths per 100,000 births per year;^11^ and inequalities in mortality amendable to health care interventions decreased by 35 deaths per 100,000 for men and 16 deaths per 100,000 for women.^44^

These more recent examples provide further evidence of the role of public policies in breaking the curve and reducing health inequalities. We can learn from these and previous historical periods to develope health-promoting policies for the twenty-first century.

## Conclusion

This article has examined historical trends in health inequalities over the 20th and 21st centuries. Drawing on studies from the United States, United Kingdom, Sweden, and Western Europe, it has concluded that, as with income and wealth inequalities, the available evidence suggests that there is a u-shaped curve in (relative) health inequalities. Taking a long view, the article argues that health inequalities generally decreased across the twentieth century through to the 1980s when they started to increase, with an additional spike since the 2010s particularly in the United Kingdom and the United States. These trends in health inequalities broadly parallel the trends identified by Piketty with regard to the u-shaped curve of income and wealth inequalities across the 20th and 21st centuries.^1^ The article sets out four distinct policy periods in the evolution of health inequalities (Interbellum Era 1920–1950; Trente Glorieuse 1950–1980; Neoliberalism 1980–2010; and Crisis Age 2010–present), which, together, provide evidence that social policies, health care access, and political incorporation have driven changes in trends over time.^36^ More recent examples of breaking the curve in Germany and England also emphasize the importance of politics and policy for health improvement.^26^

However, the u-shaped curve of health inequalities set out here is very much a working hypothesis and it requires more extensive and systematic historical analysis to fully interrogate it. For example, the sparse historical data available (particularly for pre-1960s) means that more extensive, time series historical data needs to be collated and analyzed to fully assess trends over time in different countries and for different outcomes (something which the European Cooperation in Science and Technology's initiative “The Great Leap. Multidisciplinary Approaches to Health Inequalities, 1800–2022” may enable;^45^). This article has also not used systematic review methods, so there may be articles and data which do not support the u-shaped curve hypothesis. Further, the analysis of different health outcomes (e.g., IMRs, preventable mortality, and life expectancy) for different countries and time periods is also a limitation as are the various ways in which inequality has been measured across the included historical studies (e.g., race/ethnicity, in/out of wedlock children, income, deprivation and education). Indeed, the curve appears to be more evident for relative compared to absolute inequalities.^13^
